# Forecasting the incidence of acute haemorrhagic conjunctivitis in Chongqing: a time series analysis

**DOI:** 10.1017/S095026882000182X

**Published:** 2020-08-18

**Authors:** Hongfang Qiu, Dewei Zeng, Jing Yi, Hua Zhu, Ling Hu, Dan Jing, Mengliang Ye

**Affiliations:** 1 Department of Epidemiology and Health Statistics, School of Public Health and Management, Chongqing Medical University, Chongqing 400016, China; 2 Nanan District Center for Disease Control and Prevention, Chongqing 400066, China

**Keywords:** Acute haemorrhagic conjunctivitis, Holt-Winters exponential smoothing, mean absolute percentage error, mean square error, seasonal autoregressive integrated moving average

## Abstract

Acute haemorrhagic conjunctivitis is a highly contagious eye disease, the prediction of acute haemorrhagic conjunctivitis is very important to prevent and grasp its development trend. We use the exponential smoothing model and the seasonal autoregressive integrated moving average (SARIMA) model to analyse and predict. The monthly incidence data from 2004 to 2017 were used to fit two models, the actual incidence of acute haemorrhagic conjunctivitis in 2018 was used to validate the model. Finally, the prediction effect of exponential smoothing is best, the mean square error and the mean absolute percentage error were 0.0152 and 0.1871, respectively. In addition, the incidence of acute haemorrhagic conjunctivitis in Chongqing had a seasonal trend characteristic, with the peak period from June to September each year.

## Introduction

Acute haemorrhagic conjunctivitis (AHC) is a highly infective eye disease, which is mainly caused by either enterovirus 70 (EV70) or coxsackievirus A24 (CVA24) infection [1, 2]. The main symptoms are conjunctival congestion, burning feeling, photophobia, tears, feeling foreign body and so on. Outbreaks have been found in many countries, such as India, Senegal and so on [3–5]. Although cases occur every month, AHC incidence has obvious seasonal characteristics [6]. The symptoms appear within 2 days after direct contact with the source of infection [7], and the incidence of AHC is related to temperature and humidity [8].

In China, AHC is defined as a notifiable infectious disease [9]. It is a common eye infection disease in our country and has been reported in many cities, the incidence of AHC in Chongqing ranks the top 5 in China [10]. The epidemic circumstance still serious because AHC is highly contagious and there is no specific effective treatment. Modelling and forecasting of the incidence of AHC provide a basis for developing policies and interventions.

Time series analysis is a scientific quantitative prediction of the future trend of diseases based on historical data and time variables. It is a quantitative analysis method without considering the influence of complex factors [11] and widely used in various fields. The exponential smoothing method has the characteristics of simple calculation, no requirements for data distribution, and easy operation. It is a simple and feasible short-term time series prediction method [12]. ARIMA model is one of the most representative and widely applied models in the prediction of time series [13], this method is very simple and requires only endogenous variables instead of other exogenous variables.

Although there are some AHC studies based on epidemic disease [2, 14, 15], it is the first time to apply exponential smoothing method and SARIMA model for the incidence of AHC in Chongqing. This study used monthly incidence of AHC from 2004 to 2017 as a training set for fitting SARIMA model and Holt-Winters exponential smoothing model. Monthly data of 2018 as the verification set, comparing two models of the incidence of AHC to predict performance, to determine the optimal model to predict the tendency of the incidence of AHC and provide scientific basis for AHC prevention and control in Chongqing.

## Materials and methods

### Study area and data collection

Chongqing is the fourth city firsthand under the jurisdiction of the Chinese central government， it is the linkage between developed East and central [16]. Chongqing (28°10′–32°13′N, 105°11′–110°11E) covers a land area of 82 402 km^2^ with 38 districts and counties and an inhabitant population of about 31.01 million by the end of 2018. Now, Chongqing has become the largest and most populous city in China [17].

The incidence data are primarily gained from the Chongqing Center of Disease and Control (2004–2018), the data included not only the monthly incidence of AHC but also the age, sex and occupation for each case.

### Statistical analysis

Firstly, in this study, using epidemiology to briefly describe the AHC epidemic in Chongqing, including the temporal and spatial distribution, high-incidence age group and so on. Then all the monthly incidence of AHC data were modelled and predicted, all the data analyses were performed using R 3.5.0.

### Exponential smoothing model construction

For the deterministic analysis of the series, consider the seasonality of infectious diseases, we adopted the Holt-Winters exponential smoothing model. The model structure is as follows [18]:

The additive Holt-Winters' seasonal exponential smoothing model is:
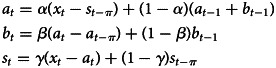


The multiplicative Holt-Winters' seasonal exponential smoothing model is:
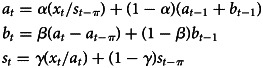


In the equation, *a*_*t*_ is the horizontal part of the sequence, *b*_*t*_ is the trend part of the sequence, *s*_*t*_ is the seasonal factor of the sequence (*π* is the cycle length of the sequence, and the cycle length studied in this paper is 12,*π* = 12).

### SARIMA model construction

For the randomness analysis of the series, given the seasonal trend, we selected the SARIMA model. According to the literatures, the SARIMA model is developed from the ARIMA model. It uses autoregressive parameters, moving average parameters and the number of differencing passes to describe a series in which a pattern is repeated over time. In this study, SARIMA model was fitted using monthly incidence of AHC as the dependent variable and its past observations as the independent variable. The general model form of SARIMA fitted to the original observation sequence is [19]：
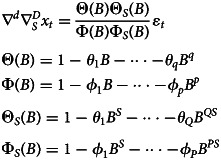


In the equation, *B* is the backward shift operator, ɛ_*t*_ is the estimated residual at time *t* with zero mean and constant variance and *x*_*t*_ denotes the observed value at time *t* (*t* = 1, 2 … *k*). The process is called SARIMA(*p*, *d*, *q*) × (*P*, *D*, *Q*)_*S*_ (*s* is the length of the seasonal period). Where *p*, *d* and *q* are the autoregressive order, number of difference and moving average order, respectively; *P*, *D* and *Q* are the seasonal autoregressive order, number of seasonal difference and seasonal moving average order, respectively [19]. According to the autocorrelation function and partial autocorrelation function of the sequence, the values of the six parameters in the SARIMA model are determined and the fitted SARIMA model is obtained. The model was optimised by comparing Akaike information criterion (AIC), smaller AIC indicate the better fitting model [20].

The SARIMA model was performed using R software, in addition, with a two-sided significance level of *P* < 0.05.

### Model comparison

The predicted accuracy of the Holt-Winters exponential smoothing model and the SARIMA model was compared by calculating the prediction error: the mean square error (MSE) and mean absolute percentage error (MAPE) [21]. Their mathematical formulas are [22]:
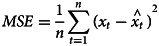

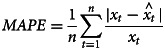


Where *x*_*t*_ is the actual incidence value, 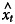 is the estimated incidence value, and *n* is the amount of months for forecasting. The MSE and MAPE were calculated to evaluate the accuracy of the forecast and to choose the best model. A lower MAPE value indicates a better fit of the data.

## Results

### Descriptive analyses

This study reported 30 686 AHC cases in the past 15 years (2004–2018), in Chongqing, including 18 121 males and 12 565 females, and a male-to-female ratio of 1.44:1. AHC mostly occur within the ages of 10–19 years, what is more, the age group of 10–19 accounted for the 62.69% of all reported cases. The highest percentage of AHC cases was found in students, amount to 0.3975 (*n* = 12 198), followed by farmers and children.

**Fig. 1.**
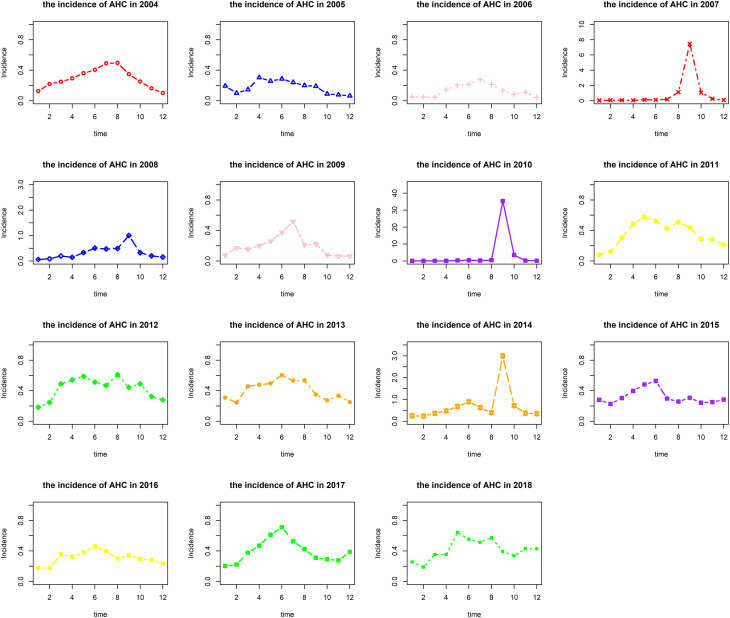
The variation of the reported incidence of AHC (1/100 000) in each year after being disintegrated according to different years.

Figure 1 shows a sequence chart of the incidence of AHC from 2004 to 2018, which is mainly manifested as the epidemic peak from June to September, it shows obvious seasonal characteristics. Furthermore, there were several outliers in 2007, 2008, 2010 and 2014.

Table 1 shows AHC ranked the top 10 regions in terms of prevalence in Chongqing from 2004 to 2018. The main popular areas of AHC were located in Kaizhou District, Beibei District and Rongchang District of Chongqing.

**Table 1.** Geographical distribution and annual incidence of AHC (1/100 000) in Chongqing from 2004 to 2018

District and County	2004	2005	2006	2007	2008	2009	2010	2011	2012	2013	2014	2015	2016	2017	2018
Kaizhou District	9.74	3.79	1.08	4.91	5.96	4.16	191.66	23.36	34.69	32.32	45.21	28.25	23.83	24.51	17.87
Beibei District	43.92	10.03	4.54	93.00	19.20	6.96	68.00	11.02	12.85	9.45	8.67	5.16	4.32	6.17	7.45
Rongchang District	–	0.17	–	6.52	8.51	10.25	130.82	12.70	20.55	19.53	15.61	5.26	7.56	22.16	31.69
Nanan District	3.43	1.29	1.09	8.91	1.09	1.26	35.88	4.34	2.49	1.71	99.41	13.98	18.65	23.41	24.58
Changshou county	3.54	1.49	0.50	13.46	0.99	1.47	118.35	4.42	2.54	1.75	2.73	2.46	2.67	1.68	1.31
Liangping County	2.72	1.79	1.02	4.07	0.89	2.27	63.28	3.49	2.85	3.65	3.88	0.83	1.78	24.51	17.87
Banan District	0.47	–	0.61	29.98	17.72	8.10	19.39	4.14	4.84	6.72	14.63	6.63	3.88	7.48	7.22
Yubei District	0.61	0.91	1.18	5.35	8.71	7.88	61.85	2.53	1.33	1.77	4.26	2.19	5.64	3.90	12.74
Qianjiang District	2.38	3.00	4.56	2.53	1.01	1.51	50.13	8.99	8.75	7.63	4.69	6.65	6.49	5.78	6.70
Wanzhou District	2.60	4.46	0.60	12.58	5.65	6.26	59.60	3.13	2.73	3.97	2.44	2.42	2.99	7.51	3.55

### Exponential smoothing model

**Fig. 2.**
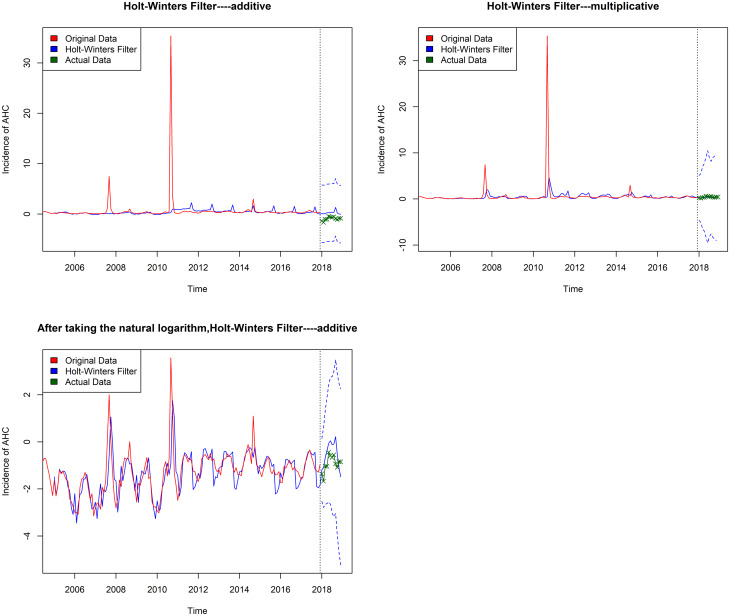
Three exponential smoothing models were used to extrapolate the monthly reported morbidity from January to December 2018 in Chongqing.

Given the season and trend effect, the data were fitted by the additive Holt-Winters' seasonal exponential smoothing model, the multiplicative Holt-Winters' seasonal exponential smoothing model, and considering several outliers, we also fitted the additive Holt-Winters' seasonal exponential smoothing model based on the original data after taking the natural logarithm. We calculate the MSE of multiplicative Holt-Winters' seasonal exponential smoothing model is 0.0152, MAPE is 0.1871 (Table 2), while MSE of additive Holt-Winters' seasonal exponential smoothing model is 0.1396, MAPE is 0.7607, and MSE of the processed additive Holt-Winters' seasonal exponential smoothing model is 0.1328, MAPE is 0.7214. A lower MSE and MAPE value indicates a better fit of the model, so the multiplicative Holt-Winters' seasonal exponential smoothing model is the optimum model in the three prediction models, and the three fitting charts were drawn in Figure 2 (the red line represents the original data, the solid blue line represents the fitting value, the dotted blue line represents the 95% confidence interval and the green line represents the actual incidence in 2018).

**Table 2.** Predictive value of 2018 incidence of AHC (1/100 000) based on the three exponentially smooth models

Time(month)	Observed value	Forecast AHC incidence		
		Model one	Model two	Model three
January	0.2569	0.0788	0.2337	0.3044
February	0.1886	0.0312	0.2132	0.3370
March	0.3544	0.1090	0.3222	0.5198
April	0.3544	0.2403	0.3403	0.6928
May	0.6439	0.2460	0.4078	0.9432
June	0.5528	0.2870	0.4721	1.0508
July	0.5138	0.3156	0.3608	0.8803
August	0.5756	0.3279	0.3024	0.9099
September	0.3935	1.3254	0.3253	1.2497
October	0.3382	0.1968	0.3253	0.5829
November	0.4292	0.0187	0.3245	0.3295
December	0.4292	0.0308	0.3636	0.2264
MSE	–	0.1396	0.0152	0.1328
MAPE	–	0.7607	0.1871	0.7214

**Fig. 3.**
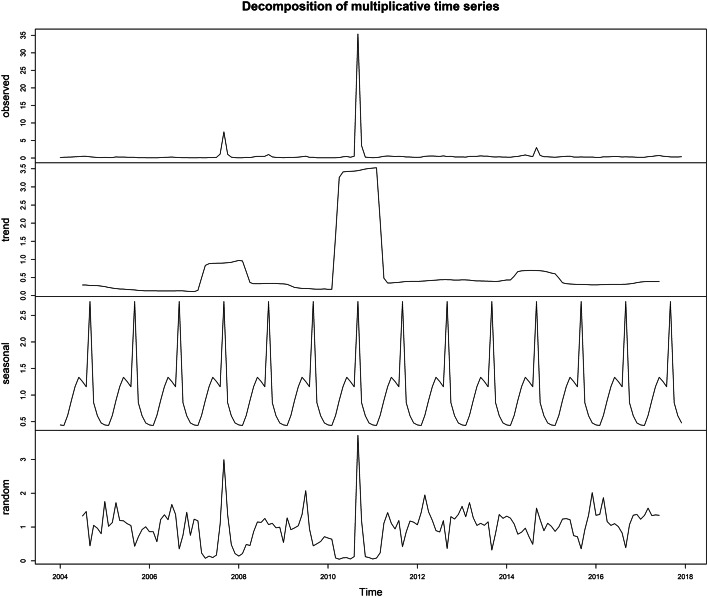
Monthly data cases of AHC incidence from 2004 to 2017 with multiplicative decomposition of AHC incidence time series data.

Table 2 shows that the predicted values by three exponential smoothing methods (model one is additive Holt-Winters' seasonal exponential smoothing method, model two is multiplicative Holt-Winters' seasonal exponential smoothing method, model three is the additive Holt-Winters' seasonal exponential smoothing method after taking the natural logarithm of the original data). In addition, we used the ‘decompose’ function to decompose sequences. As shown in Figure 3, the data have obvious seasonality, tendency, and the residual sequence diagram is stationary, the multiplicative Holt-Winters' seasonal exponential smoothing model is significant.

### SARIMA model

**Fig. 4.**
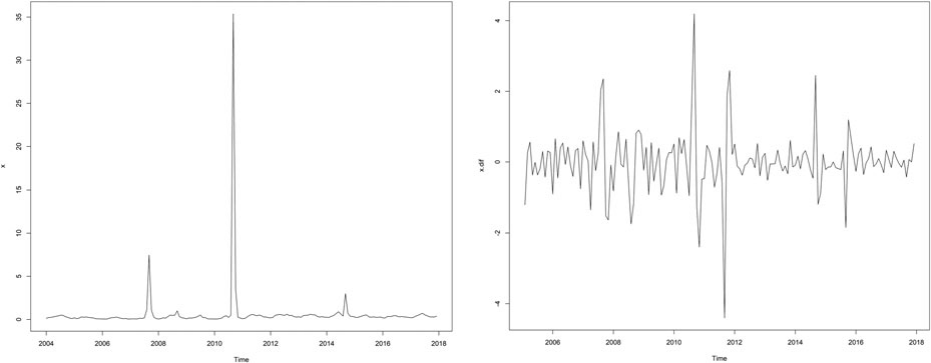
Time series plot of the incidence of AHC from January 2004 to December 2017, in Chongqing.

Firstly, a sequence diagram was drawn for AHC incidence data (Fig. 4a), the sequence diagram is non-stationary. After the natural logarithm transformation of the original data, we conducted a one-step difference and seasonal difference with a period of 12 to remove seasonal trends. The sequence diagram after the difference is basically stationary (Fig. 4b), and the ADF test results show that the sequence is stationary (*P* < 0.05).

**Fig. 5.**
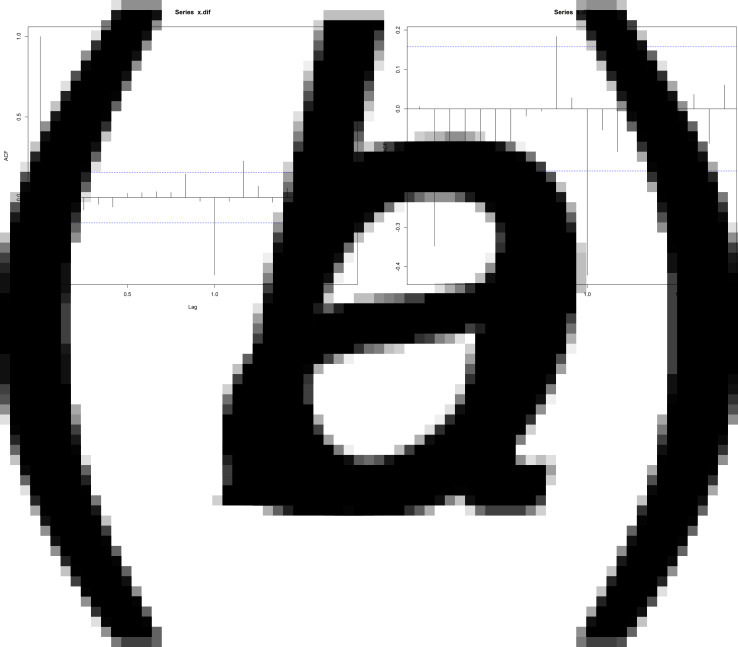
Autocorrelation function plot (a) and partial autocorrelation function plot (b) after first differentiation.

We estimated the six parameter values of the SARIMA (*p*, *d*, *q* and *P*, *D*, *Q*) model based on the ACF and PACF graphs of the transformed time series. Observed the graphs of ACF and the graphs of PACF (Fig. 5) after data transformation, we developed four models and chosen ‘CSS-ML’ method to estimate the parameters.

### SARIMA (1, 1, 1) (0, 1, 1) _12_

Table 3 shows AIC values, MSE and MAPE for the SARIMA models to various choices of *p* and *q*. Although the MSE and MAPE values of the four model training sets are very close, the SARIMA(1, 1, 2) × (0, 1, 1)_12_ model had the lowest AIC value. Goodness-of-fit analysis indicated that the SARIMA(1, 1, 2) × (0, 1, 1)_12_ model fitted the data reasonably well. Moreover, in the residual white noise test of SARIMA(1, 1, 2) × (0, 1, 1)_12_ model, the *P*-values of LB statistics were 0.9488 and 0.9981 (*P* > 0.05), respectively. The residual sequences are pure random, the stationary residual sequence indicates that the fitted SARIMA(1, 1, 2) × (0, 1, 1)_12_model is sufficient, the SARIMA(1, 1, 2) × (0, 1, 1)_12_ model was significant. The equation of the SARIMA was:
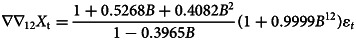

**Table 3.** Comparisons of the tested SARIMA models for the incidence of AHC in Chongqing

Model	AIC	MSE	MAPE
SARIMA(1, 1, 2)(0, 1, 1)_12_	287.6	0.2591	0.9595
SARIMA(1, 1, 2)(1, 1, 1)_12_	289.54	0.2596	0.9168
SARIMA(2, 1, 2)(0, 1, 1)_12_	288.62	0.2559	0.9741
SARIMA(2, 1, 2)(1, 1, 1)_12_	290.58	0.2562	0.9340

**Fig. 6.**
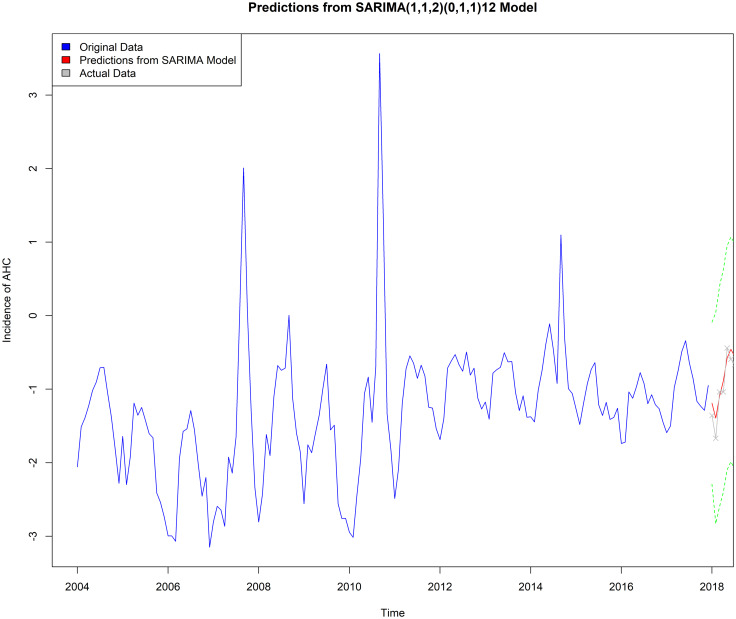
The trend plot of the forecast using the SARIMA(1, 1, 2) × (0, 1, 1)_12_ model.

The predicted values were verified by applying the value from January to December 2018. The actual incidence and forecasted incidence from January to December 2018 in the Table 4 and the prediction diagram is shown in Figure 6 (the blue line represents the actual value, the solid red line represents the predicted value, the dashed red line represents the 95% confidence interval, and the solid grey line represents the actual value). It demonstrated that the results show that the predicted value can fluctuate up and down with the original series. The MSE of prediction was 0.03814 and the MAPE of prediction was 0.3164, respectively. Furthermore, some outliers appeared in the predicted value, for example, the predicted incidence of AHC in September 2018 was 0.9946/100 000, while the actual incidence was 0.3935/100 000. Showing a significant difference, which may be caused by particularly high values in 2007, 2008, 2010 and 2014 year, such as the incidence of AHC was 35.3725/100 000 in September 2010.

**Table 4.** Prediction of the incidence of AHC (1/100 000) in 2018 based on SARIMA model

Month	Observed value	Predicted value	95% CI
January	0.2569	0.3041	0.1035–0.8938
February	0.1886	0.2481	0.0606–1.0164
March	0.3544	0.3346	0.0771–1.4520
April	0.3544	0.4131	0.0936–1.8228
May	0.6439	0.5627	0.1265–2.5022
June	0.5528	0.6334	0.1417–2.8319
July	0.5138	0.5721	0.1274–2.5696
August	0.5756	0.5956	0.1321–2.6866
September	0.3935	0.9946	0.2196–4.5049
October	0.3382	0.4738	0.1042–2.1553
November	0.4292	0.3107	0.0680–1.4194
December	0.4292	0.2358	0.0514–1.0820

Using SARIMA(1, 1, 2) × (0, 1, 1)_12_model and exponential smoothing Holt-Winters with multiplicative seasonality model to predict the incidence of AHC in 2018, the MSE and MAPE values of the two models were calculated in Table 5, and the comparison showed that the prediction effect of the exponential smoothing Holt-Winters with multiplicative seasonality model was slightly better than that of the SARIMA(1, 1, 2) × (0, 1, 1)_12_model.

**Table 5.** The prediction accuracy of SARIMA(1, 1, 2) × (0, 1, 1)_12_ model was compared with that of Holt-Winters multiplicative model.

	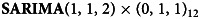	Holt-Winters multiplication model
MSE	0.03814	0.0152
MAPE	0.3164	0.1871

## Discussion

AHC is one of the common eye diseases in Chongqing. The annual incidence was 1.569/100 000 at the lowest and 41.1682/100 000 at the highest from 2004 to 2018, in Chongqing, and affects nearly 1.5 times as many males as females in a total of 30 686 reported cases (1.44:1), which is consistent with some previous research results [23]. The high peaks about age and career of AHC cases were from 10 to 19 years old (62.69%) and students (39.75%), respectively, which indicate that the important protection objectives should be concentrated in students and teenagers. Moreover, although AHC occurs all year, the peak of the incidence of AHC is from June to September each year, showing the obvious periodicity and seasonality [24]. In this study, there are several special values that occurred in 2007, 2008, 2010 and 2014, respectively, which may be related to frequent flood disasters in Chongqing, especially the severe and rare floods that occurred in Chongqing in 2007 [25]. According to literature reports, AHC is most likely to break out and spread after flood. The destruction and pollution of water source and related living infrastructure, the obvious decline of sanitary conditions, and the unstable psychological factors of the population after flood, which may lead to the decline of immunity [26, 27]. Furthermore, Kaizhou District, Beibei District and Rongchang District are high-attack areas in Chongqing. This may be relevant to different geographical features and demographic structure, and lack of education promotion, so we should control AHC reasonably according to geographical differences. So before the high-incidence season comes, strengthen the health management of key public places and improve the health system. During the epidemic season, hospitals should start specialised temporary outpatient clinics, strengthen the pre-screening and triage of patients, pay attention to disinfection of ophthalmic devices, prevent cross-infection. At the same time, strengthen education and publicity, wash hands frequently, and do a good job in personal and family hygiene and so on.

In this paper, we performed time series analysis on the data from two aspects. Deterministic analysis, we adopted exponential smoothing method to analyse. It has been generally applied in economy, industry, agriculture, transportation, medicine [28]. The other is stochastic analysis, we adopted SARIMA model. ARIMA model has been more and more applied to illustrate the time patterns of malaria [29], tuberculosis [30, 31], dengue [32] and other diseases [33, 34].

According to the sequence diagram of AHC incidence, there is obvious seasonality and tendency in this sequence. Therefore, we use the Holt-Winters exponential smoothing method for analysis. The exponential smoothing Holt-Winters with additive seasonality model and exponential smoothing Holt-Winters with multiplicative seasonality model were established. At the same time, considering that there are some high values in the original sequence, the original data were transformed into the natural logarithm, while the sequence after the natural logarithm transformation can only be transformed into the additive Holt-Winters' seasonal exponential smoothing model. Then the predictive value and prediction graph of the incidence of AHC by three exponential smoothing models were calculated, and the comparison showed that the multiplicative Holt-Winters' seasonal exponential smoothing method had the best predictive effect.

For the random analysis of non-stationary sequences, considering the existence of seasonal factors and trend factors, this paper adopted the SARIMA model for modelling and analysed the four SARIMA models based on the incidence of AHC. By comparing the AIC values of the four models, it was concluded that the SARIMA(1, 1, 2) × (0, 1, 1)_12_ model was the best.

Finally, in order to compare the prediction effect of the Holt-Winters multiplicative exponential smoothing model and the SARIMA(1, 1, 2) × (0, 1, 1)_12_ model, the MSE and MAPE values of the two models were calculated respectively in this paper. The results showed that the predictive effect of the multiplicative Holt-Winters' seasonal exponential smoothing model was slightly better than that of the SARIMA(1, 1, 2) × (0, 1, 1)_12_ model. Therefore, it follows that the exponential smoothing Holt-Winters with multiplicative seasonality model was better for short-term prediction of the incidence of AHC. Moreover, the incidence of AHC showed significant seasonality, the exponential smoothing Holt-Winters with multiplicative seasonality model might be suitable for the forecast of time-series data with trends and seasonality in this paper.

There are some limitations of this study, in this study, only the incidence of AHC was used for preliminary modelling prediction, and many factors affecting the incidence of AHC were not considered in this paper, in addition, we only made short-term predictions. Therefore, in order to establish a long-term stable prediction model, various factors affecting the incidence of AHC need to be considered. In the following research work, we can use hybrid models to analyse or predict disease, such as SARIMA-NAR hybrid model [22], SARIMA-NARNNX hybrid model [35], SARIMA-NARX hybrid model [36] and so on.

## Conclusions

The short-term forecast of AHC can evaluate the prevention or control measures. Meanwhile, we can adopt timely and effective countermeasures for the epidemic peak that may occur. In this paper, the exponential smoothing based on the multiplicative model could be availably used in the time series analysis of AHC in Chongqing, and all the actual values fell in the confidence interval of the predicted value. It suggests that exponential smoothing can be used to predict the incidence of AHC. So the predictions of the incidence of AHC could generate useful information for designing the strategies of public health services.

## Data Availability

The incidence of AHC data is gained from the Chongqing Center of Disease and Control, it is confidential data and cannot be uploaded to your organisation. The incidence is equal to the number of new cases of a disease in a population during a period divided by the number of people exposed during the same period.
